# Goat Cheese Produced with Sunflower (*Helianthus annuus* L.) Seed Extract and a Native Culture of *Limosilactobacillus mucosae*: Characterization and Probiotic Survival

**DOI:** 10.3390/foods13182905

**Published:** 2024-09-13

**Authors:** Dôrian Cordeiro Lima Júnior, Viviane Maria da Silva Quirino, Alícia Santos de Moura, Joyceana Oliveira Correia, João Ricardo Furtado, Isanna Menezes Florêncio, Márcia Maria Cândido da Silva, Hévila Oliveira Salles, Karina Maria Olbrich dos Santos, Antonio Silvio do Egito, Flávia Carolina Alonso Buriti

**Affiliations:** 1 Programa de Pós-Graduação em Ciências Farmacêuticas, Universidade Estadual da Paraíba, R. Juvêncio Arruda, s/n, Campina Grande 58429-600, PB, Brazil; mrjuniorc7@gmail.com (D.C.L.J.); joyceanaoliveira@gmail.com (J.O.C.); flavia@servidor.uepb.edu.br (F.C.A.B.); 2 Núcleo de Pesquisa e Extensão em Alimentos, Universidade Estadual da Paraíba, R. Juvêncio Arruda, s/n, Campina Grande 58429-600, PB, Brazil; qviviane9@gmail.com (V.M.d.S.Q.); aliciamoura1998@gmail.com (A.S.d.M.); isannamenezes@hotmail.com (I.M.F.); 3 Embrapa Caprinos e Ovinos, Estrada Sobral/Groaíras, km 4, Sobral 62010-970, CE, Brazil; joao.furtado@embrapa.br (J.R.F.); hevila.salles@embrapa.br (H.O.S.); 4 Embrapa Caprinos e Ovinos, Núcleo Regional Nordeste, R. Osvaldo Cruz, 1143, Campina Grande 58428-095, PB, Brazil; marciamcandido@gmail.com; 5 Embrapa Agroindústria de Alimentos, Av. das Américas, 29501, Rio de Janeiro 23020-470, RJ, Brazil; karinal.dos-santos@embrapa.br

**Keywords:** biopreservative effect, goat dairy product, vegetable rennet cheese, potentially probiotic autochthonous culture

## Abstract

The microbiological and biochemical properties of a goat cheese produced using *Helianthus annuus* (sunflower) seed extract as a coagulant and the potentially probiotic autochthonous culture *Limosilactobacillus mucosae* CNPC007 were examined in comparison to a control cheese devoid of the autochthonous culture. Throughout a 60-day storage period at 6 ± 1 °C, lactobacilli maintained a count of above 8 log CFU/g. Additionally, its viability in cheeses subjected to the in vitro gastrointestinal conditions demonstrated improvement over this period. Specifically, the recovery of lactobacilli above 6 log CFU/g was observed in 16.66% of the samples in the first day, increasing to 66.66% at both 30 and 60 days. While total coliforms were detected in both cheese trials, this sanitary parameter exhibited a decline in *L. mucosae* cheeses during storage, falling below the method threshold (<3 MPN/g) at 60 days. This observation suggests a potential biopreservative effect exerted by this microorganism, likely attributed to the higher acidity of *L. mucosae* cheeses at that point (1.80 g/100 g), which was twice that of the control trial (0.97 g/100 g). Furthermore, distinct relative proportions of >30 kDa, 30–20 kDa, and <20 kDa proteins during storage was verified for *L. mucosae* and control cheeses. Consequently, either the *H. annuus* seed extract or the *L. mucosae* CNPC007 autochthonous culture influenced the biochemical properties of the cheese, particularly in terms of proteolysis. Moreover, *L. mucosae* CNPC007 acidification property resulted in a biopreservative effect throughout the storage period, indicating the potential as a promising source of probiotics for this product.

## 1. Introduction

Goat milk, bearing a profile more akin to human milk, as opposed to cow and sheep milk, offers a plethora of health advantages to consumers, such as a reduced risk of eliciting allergic reactions in individuals with cow milk allergies [1,2,3], heightened digestibility and alkalinity, as well as enhanced therapeutic and nutritional attributes [4,5,6]. Such distinguishing features position goat milk as an outstanding foundation for the manufacture of various food products, including cheeses [7].

In the context of goat cheese production, the utilization of milk-clotting enzymes derived from plant sources presents an intriguing alternative to the rennet from animal and microbial origin [8,9,10]. This choice is driven by the renewability of plant inputs and the presence of these enzymes in various species within the plant kingdom [11,12,13]. Notably, it is essential to underscore that numerous cheeses worldwide are crafted using plant extracts, particularly in countries such as Spain, Portugal, Italy, France, Middle Eastern nations, and African countries [14,15,16].

The milk-clotting potential of *Helianthus annuus* (sunflower) seed extract has been described in various studies [17,18]. Egito et al. [17] reported on the milk-clotting capabilities of *H. annuus* extract in cow milk. Nasr et al. [18] demonstrated that *H. annuus* extract represents an economical milk-clotting method suitable for cheese production using both cow and goat milk, thanks to its robust rennet- and curd-forming properties. The main milk-clotting enzyme reported for the sunflower seeds is the aspartic proteinase [17,18,19,20,21]. Moreover, in a study conducted by Giada [22], an aqueous extract of *H. annuus* seeds was investigated, revealing that phenolic compounds constituted approximately 1.34% of the samples, a noteworthy proportion. Within this group of phenolic compounds, chlorogenic acid, quininic acid, caffeic acid, and p-hydroxybenzoic acid were identified [23,24,25]. This distinctive attribute, coupled with its application in goat cheese production, presents an enticing opportunity for the development of functional foods.

This is reinforced by the growing demand among the population for functional foods that offer benefits beyond their nutritional value, which was amplified during the COVID-19 pandemic, where there was a significant interest in methods that can strengthen the immune system and boost antioxidant mechanisms, such as plant-derived supplements and probiotic foods [26,27]. In this context, the development of new probiotic goat cheese enriched with plant-based ingredients aims to meet the current demands of consumers who are looking for such products. Particularly for clotted cheeses using a plant extract that is a source of proteases, it is necessary to evaluate whether the sunflower extract has any influence on the proteolytic activity in this type of product throughout storage.

The quest to augment the array of probiotic strains takes on pivotal significance, particularly in developing nations, where access to probiotic-rich foods remains restricted to small-scale dairy producers [28]. Within the *Lactobacillaceae* family, multiple potentially probiotic species can be found, among which *Limosilactobacillus mucosae* (formerly *Lactobacillus mucosae*) stands out. This bacterium exhibits proficient intestinal colonization, capabilities attributed to its adhesion to the gastrointestinal epithelium. Notably, *L. mucosae* has demonstrated the ability to modulate the intestinal immune system and inhibit pathogenic bacteria, rendering it an invaluable asset in the realm of probiotic food development [29,30]. The autochthonous strain *Limosilactobacillus mucosae* CNPC007 (formerly *Lactobacillus mucosae* CNPC007), initially isolated from goat milk by a team of researchers at the Brazilian Agricultural Research Corporation (EMBRAPA), exhibited the presence of genetic factors related to intestinal adhesion properties (*msa*, *map*, *mub*, and *ef-tu*) and to bile salt hydrolase production (*bsh*), the ability to deconjugate salts of glycodeoxycholic acid, and a high survival rate in simulated gastric and enteric in vitro conditions, properties that render this strain as potential probiotic candidate [30]. Moreover, this strain also showed a relatively high milk acidification and clotting capacity, diacetyl production, and proteolytic activity [16], that enforced it to have undergone comprehensive study and application in various food products, including goat cheese [31]. Therefore, it is desirable to investigate if this microorganism is able to develop proteolytic activity in a plant rennet goat cheese and if this food matrix is able to preserve the survival of this culture under in vitro simulated gastrointestinal conditions.

Furthermore, the potential of lactic acid bacteria, including probiotic *Lactobacillaceae* strains, as biopreservative agents in food has been recognized over the years due to their capacity as lactic acid producers, creating a hostile environment for spoilage and pathogenic bacteria. Some lactic acid bacteria also exhibit proteolytic activity, releasing bioactive peptides, and can produce bacteriocins. The biopreservative effect arises from the competitive growth and generation of antimicrobial molecules by lactic acid bacteria through their metabolic processes, fostering a natural or controlled microbiota. This, in turn, extends the shelf life and enhances the safety of products [32,33]. Recently, Galdino et al. [33] confirmed that *L. mucosae* CNPC007 could inhibit standard strains of sanitary indicators such as *Salmonella enterica* serovar Typhimurium ATCC 14028, *Staphylococcus aureus* ATCC 25923, and *Escherichia coli* ATCC 25922 in vitro. These characteristics could heighten the appeal of producing plant-rennet goat cheese with probiotic and biopreservative potential.

Considering these opportunities, the concurrent utilization of goat’s milk, plant extract and an autochthonous potentially probiotic microorganism in cheese production may enable the creation of a top-tier food product with multiple functional health benefits. Additionally, it can offer a novel food alternative for local producers to meet the increasing market demand for functional foods among the general population in recent years.

The objective of this study was to assess the composition, the biopreservative potential against microbial sanitary indicators and the proteolysis profile of a goat cheese manufactured using *Helianthus annuus* (sunflower) seed extract and the potentially probiotic autochthonous culture of *Limosilactobacillus mucosae* CNPC007. This assessment was conducted by comparing it with a control cheese produced without the potentially probiotic culture. Moreover, to explore the viability of this food product as a carrier for *L. mucosae* CNPC007 as a probiotic over a 60-day storage period, the cheese containing this microorganism underwent an in vitro assay simulating the conditions of the gastrointestinal tract at 30-day intervals.

## 2. Materials and Methods

### 2.1. Helianthus Annuus (Sunflower) Seed Extract

The seed extract was prepared following the methodology outlined by Egito et al. [7], with some modifications. *Helianthus annuus* seeds were acquired from the local market in Campina Grande, Paraíba State, Brazil. Initially, the seeds underwent a one-hour soak in a 1% sodium hypochlorite solution. Subsequently, they were thoroughly rinsed with distilled water to eliminate any residual sodium hypochlorite. Next, 1.2 kg of sunflower seeds were finely ground in a blender until a uniform mass was achieved. To this mass, 1.5 L of 1% saline solution was added and the mixture was homogenized. The resulting mixture was then refrigerated at temperatures below 5 °C for a period of 24 h. Following this incubation, liquid extraction was carried out using a cheese press. The extracted liquid was subsequently stored in polyethylene bottles (ca. 500 mL) and frozen at −18 °C until it was ready for use in cheese production.

### 2.2. Activation of the Lyophilized Strain

The strain of *L. mucosae* CNPC007, supplied in lyophilized form by the Brazilian Agricultural Research Corporation (EMBRAPA), underwent two activation cycles in De Man Rogosa and Sharpe (MRS) broth, following good microbiological practices, and each cycle was conducted at 37 °C for 24 h.

The tubes were subsequently centrifuged at 3018× *g* for 10 min using a Parsec Biotechnik, model CT 0603 (Curitiba, Brazil). The resulting pellet was washed with 10 mL of a 0.85% saline solution, and this washing process was repeated three times to yield the pellets (with an average of 3.4 × 10^10^ CFU per pellet) utilized in the cheese manufacturing process.

### 2.3. Production of Cheese Batches

**Figure 1:**
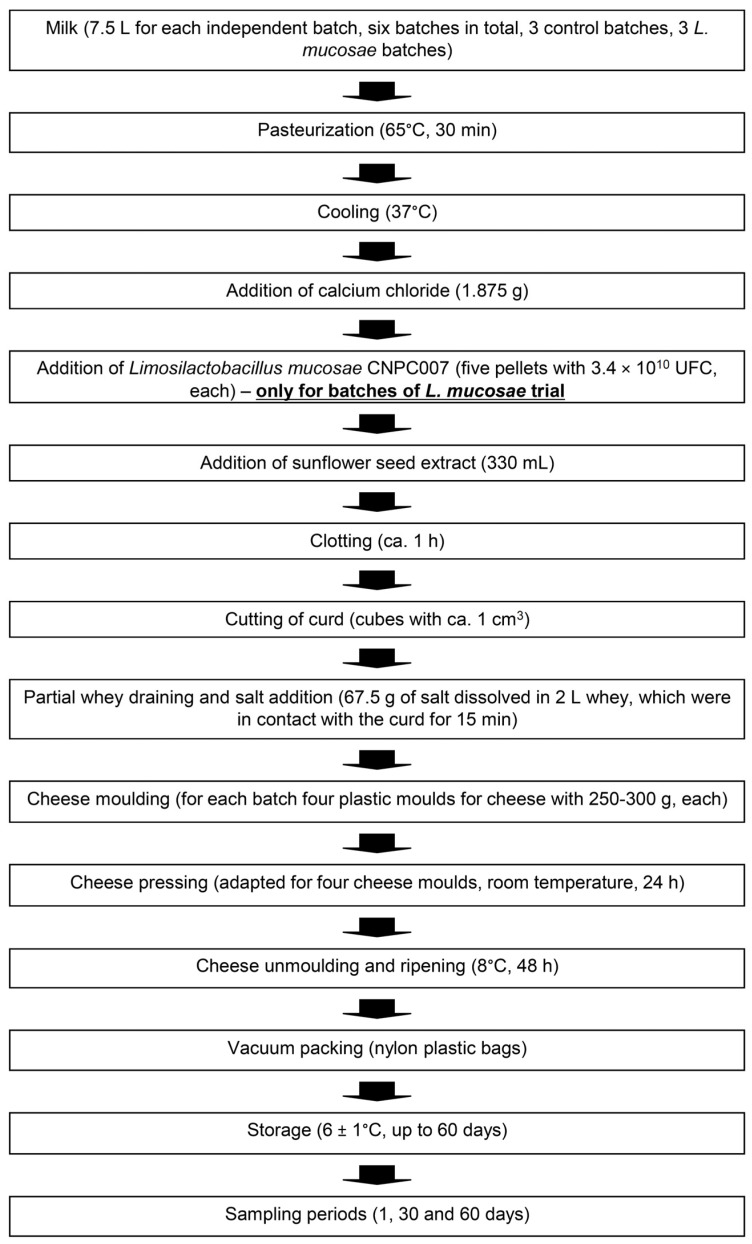
Complete cheesemaking process of the cheese trials studied.

The milk utilized in cheese production was purchased from small farmers from the municipality of Soledade, Paraíba, Brazil, and subjected to pasteurization at 65 °C for 30 min. Using milk from the same purchasing, two distinct cheese making trials were conducted; one trial incorporated the strain *L. mucosae* CNPC007, while the other, serving as the control, was produced without the inclusion of the lactic culture. Each cheese trial (control and *L. mucosae*) was repeated three times, consisting of three separate batches (genuine independent replicates). In both cheese trials, 7.5 L of goat milk at 37 °C were used, along with 330 mL of sunflower seed extract and 1.875 g of calcium chloride. For the *L. mucosae* trial, 5 pellets with the activated culture were added (1.7 × 10^11^ CFU), in order to obtain enough amount that was required to guarantee high levels of *L. mucosae* in cheese and, consequently, to maximize the survival of this probiotic through the in vitro gastrointestinal conditions. Following coagulation (ca. 1 h), the cheeses underwent partial draining, salting (67.5 g of salt was dissolved in approximately 2 L of whey, which remained in contact with the curd for 15 min), and were then placed in cheese molds. At the time of molding, the average viability of lactobacilli in the curd was 1.4 × 10^9^ CFU/g. Subsequently, they were pressed (Zatti Indústria Ltd., Coronel Freitas, Brazil) at room temperature for 24 h. Following this phase, the cheeses were removed from the molds, underwent a 48 h ripening period at 8 °C, and were finally vacuum sealed in nylon plastic bags. These vacuum-sealed cheeses were stored at 6 ± 1 °C for 60 days. Cheese samples from each trial were collected for analysis on day 1 (the day the cheese was ready for packaging after ripening), and also after 30 and 60 days of storage at 6 ± 1 °C. The complete cheese-making process for the studied cheese trials is illustrated in Figure 1.

### 2.4. Analysis of Proximate Composition and Physicochemical Parameters of Cheeses

All analyses of the proximate composition and physicochemical parameters were conducted in triplicate to ensure accuracy and reliability of the results.

The titratable acidity analysis was carried out on the cheese samples during the designated sampling periods, 1, 30, and 60 days of storage. This involved mixing 10 g of the sample with 95% ethyl alcohol to reach 100 mL of total volume and, after a 6 h interval, the mixture was filtered (185 mm qualitative paper, 80 g/m^2^, Unifil, Curitiba, Brazil). A portion of the filtrate was then titrated using a 0.1 mol/L sodium hydroxide solution, with 0.1% phenolphthalein serving as the indicator. The results are expressed as a percentage of lactic acid [34].

Except for moisture, which was also assessed during the sampling periods previously described (1, 30, and 60 days), the proximate composition analyses were conducted on the first day of storage. Moisture and total solids were determined by subjecting the samples to drying in a vacuum oven (model 104/30, Lucadema, São José do Rio Preto, Brazil), at 70 °C, until a constant weight was achieved [34]. Ash content was determined through gravimetry after incinerating the organic matter of the dried samples until complete ashed in a muffle furnace (Fornitec, São Paulo, Brazil) at 550 °C [34]. Fat content was determined utilizing a milk butyrometer following digestion of the cheese samples with H_2_SO_4_ (density = 1.5 g/mL) and isoamyl alcohol. The butyrometer, containing the digested material, was centrifuged in a Gerber centrifuge (Plurinox, Batatais, Brazil) for 10 min [34]. Protein content was estimated by quantifying the nitrogen content of 0.2 g cheese samples using the micro Kjeldahl method and then multiplying the results by 6.38 [35,36]. Total carbohydrates were calculated as the difference between 100 g of sample and the sum of the other components [36].

### 2.5. Probiotic Viability Analysis

The viability of lactobacilli was assessed throughout the *L. mucosae* cheese storage period in accordance with the previously outlined sampling schedules (1, 30, and 60 days). To achieve this, 25 g portions of the sample were aseptically transferred into 225 mL of peptone water (0.1%, *w*/*v*) and this mixture underwent serial dilutions using the same diluent [1,18]. Subsequently, 1 mL aliquots of these dilutions were pour plated onto MRS agar (HiMedia, Mumbai, India). The plates were then incubated in aerobic conditions at 37 °C for 48 h [23].

### 2.6. Resistance of the Probiotic to Passage through the Gastrointestinal System

The assessment of the resistance of lactobacilli present in *L. mucosae* cheeses under simulated gastrointestinal conditions was conducted throughout the storage period, employing the methodology as previously described by Galdino et al. [37], with some adaptations. To accomplish this, 25 g portions of the samples were diluted in 225 mL of a 0.5% sodium chloride solution. To 10 mL of these dilutions, 0.3 mL of a 1 mol/L hydrochloric acid solution and solutions of the enzymes porcine mucosa pepsin (Sigma Aldrich, St. Louis, MO, USA, 0.1 mL) and lipase (Amano lipase F-AP15, from *Rhizopus oryzae*, Sigma, 0.01 mL) in 0.5% sodium chloride solution were added, resulting in a total volume of 11.4 mL (3.07 g/L pepsin; 0.9 mg/L lipase) with a pH between 1.5 and 2.0. These dilutions with the enzyme solutions were incubated at 37 ± 1 °C for 2 h with agitation (150 rpm). Following this, aliquots of 0.17 mL of a 0.5% sodium chloride solution and 2 mL of sodium phosphate solution pH 12 (NaH_2_PO_4_2H_2_O, 14 g; 1 N NaOH, 150 mL; distilled H_2_O qsp 1 L) containing the bile (Bovine Bile, Sigma, 0.1017 g) and pancreatin (Porcine Pancreas Pancreatin, Sigma, 0.0101 g) were added, resulting in a total volume of 13.57 mL (9.53 g/L bile; 0.953 g/L of pancreatin) with a pH between 4.7 and 5.8. These sample dilutions were incubated once more at 37 ± 1 °C for 2 h with agitation at 150 rpm. Four hours after the initiation of the assay, a 1 mL aliquot of sodium phosphate solution at pH 12 containing bile (0.1448 g) and pancreatin (0.0145 g) was added, resulting in a final volume of 14.57 mL (9.57 g/L bile; 0.9570 g/L pancreatin), with a pH between 6.2 and 6.7. The sample dilutions were then incubated at 37 ± 1 °C for an additional 2 h with agitation at 150 rpm, amounting to a total assay duration of 6 h. Aliquots (1 mL) of the cheese dilutions containing the simulated gastrointestinal fluids were collected after 30 min, 2 h, 4 h, and 6 h of the in vitro assay. These aliquots were serially diluted and pour plated onto MRS agar, followed by incubation in aerobic conditions at 37 °C for 48 h. Each assay was performed in duplicate.

### 2.7. Analysis of Sanitary Conditions

In accordance with their respective classification for moisture content [38], the produced cheeses were assessed for the presence of microbiological contaminants, adhering to the regulatory standards in force at the time when the study was conducted [39].

To ascertain the presence of both total and thermotolerant coliforms, portions of the cheese samples were initially diluted in peptone water at a 1:10 ratio, following the same approach described earlier for the analysis of lactobacilli. After this, sequential dilutions were performed until a 10^−3^ dilution was achieved.

In the presumptive phase, samples from each dilution were taken and added in triplicate to tubes containing 10 mL of lauryl-tryptose broth (Kasvi, São José dos Pinhais, Brazil), along with inverted Durham tubes. These tubes were then incubated at 35 °C for 24 h. For the confirmatory phase regarding total coliforms, the samples with Durham tubes showing the presence of gas from the presumptive phase were transferred to tubes containing 10 mL of the brilliant green bile lactose broth (Kasvi, São José dos Pinhais, Brazil) also with inverted Durham tubes. These samples were incubated at 35 °C for an additional 24 h. The results from this stage were reported as the most probable number (MPN)/g of total coliforms (at 35 °C)/g of cheese. In the confirmation phase for thermotolerant coliforms, the samples that exhibited gas production in the presumptive phase were transferred to tubes containing 10 mL of EC broth culture medium (Kasvi, São José dos Pinhais, Brazil), including inverted Durham tubes. These samples were then incubated at 45 °C for 24 h. The results from this stage were presented as the MPN of thermotolerant coliforms (at 45 °C)/g of cheese.

To detect the presence of *Staphylococcus* spp., 100 µL of the decimal dilutions were spread onto Petri dishes containing mannitol salt agar (Kasvi, São José dos Pinhais, Brazil). These plates were then incubated at 37 °C for a period of 24–48 h.

To identify the presence of *Salmonella* spp., the 10^−1^ dilution, prepared as described earlier, underwent incubation at 35 °C for 24 h for enrichment. After this period, an aliquot of the enriched sample was collected using a platinum loop and streaked onto Petri dishes containing *Salmonella* differential agar (RajHans medium, HiMedia). These plates were then incubated at 35 °C for an additional 24 h.

### 2.8. Proteolytic Analysis by Sodium Dodecyl Sulphate Polyacrylamide Gel Electrophoresis (SDS-PAGE)

The PAGE-SDS-2β-mercaptoethanol system, as described by Laemmli [40] and adapted for the use of plate gels (18 × 16 cm) (SE600, GE Healthcare, Global Life Sciences Solutions, Marlborough, MA, USA), was employed. A 15% polyacrylamide separating gel and a 5% stacking gel were utilized. To prepare the SDS-PAGE gel, cheese samples (2 mg) were dissolved in Tris-HCl buffer at pH 6.8, containing SDS (1.6 mg/100 mL), 2-mercaptoethanol (4 mL/100 mL), glycerol (10 mL/100 mL), and bromophenol blue (10 mg/100 mL). This mixture was then boiled at 100 °C for 3 min. Volumes of 30 μL of the prepared samples were loaded onto the gel. Electrophoresis was conducted at 4 °C for 168 min at the following settings: 500 V, 120 mA, and 60 W. For molecular weight standards, the SigmaMarker^TM^ wide range (6500–200,000 Da, Sigma-Aldrich, Saint Louis, MO, USA) was employed, which included myosin (200 kDa), β-galactosidase (116 kDa), phosphorylase B (97 kDa), bovine serum albumin (66 kDa), glutamic dehydrogenase (55 kDa), ovalbumin (45 kDa), glyceraldehyde-3-phosphate dehydrogenase (36 kDa), carbonic anhydrase (29 kDa), trypsinogen (24 kDa), trypsin inhibitor (20 kDa), α-lactalbumin (14.2 kDa), and aprotinin (6.5 kDa). Following electrophoresis, the proteins or peptides were fixed with a 12% (*w*/*v*) trichloroacetic acid solution for 30 min and subsequently stained overnight using a solution of R-250 Coomassie blue (0.8 g/100 mL), ammonium sulphate (8 g/100 mL), phosphoric acid (0.96 mL/100 mL), and ethanol (20 mL/100 mL). The gel was then destained overnight in a solution comprising 30% (*v*/*v*) ethanol, 7.5% (*v*/*v*) acetic acid, and 5% (*w*/*v*) trichloroacetic acid. The SDS-PAGE gel was photographed after destaining and the molar masses of bands were identified by measuring their relative mobilities in comparison with those of the markers from the standard mixture processing the bands using the GelAnalyzer software, version 23.1.1, http://www.gelanalyzer.com/ (accessed on 26 July 2024).

### 2.9. Statistical Analysis

To assess the data’s homogeneity, the Levene test was utilized. For comparing data related to cheese storage time, the Friedman’s non-parametric test was employed, with a significance level of *p* < 0.05. In the case where significant results were obtained, the Wilcoxon test was used to analyze the contrasts. Additionally, for comparing the cheese trials on the same day of storage, the Mann–Whitney U test was applied. All data analyses were conducted using Statistica 9.0 Software (StatSoft®, Tulsa, OK, USA).

To assess the microbial sanitary indicators during cheese storage of cheeses and the survival of lactobacilli through the in vitro assay, the exact binomial test was carried out [41]. The null hypothesis (H_0_) posited that the presence of sanitary indicators (microbiological analysis during storage for safety evaluation) and the survival rate above 6 log CFU/g throughout the assay (in vitro simulated gastrointestinal resistance) should be at least 25%.

## 3. Results and Discussion

### 3.1. Sunflower Seed Extract Properties and Cheese Proximate Composition and Physicochemical Parameters

The figures showing the appearance and the milk clotting property of the *Helianthus annuus* seed extract processed in the present study are shown in Supplementary Figures S1 and S2, respectively (see Supplementary Data). The extracts were fluid, slightly opaque, with a brownish color (Figure S1). After being the extracts frozen and thawed for use, these characteristics were maintained. During the preliminary studies, clotting activity in tubes containing 1 mL of reconstituted milk powder and 500 µL extract was verified after 20 min, while milk in tubes containing 40 µL extract clotted after 1 h (Figure S2). Therefore, 40 µL extract for each 1 mL milk was used as reference to define the amount of extract that would be used in cheese production, with an increase of 10% in order to be used with a higher milk volume. This milk-clotting property of the extract was maintained even after being frozen and thawed.

The results of the analyses of proximate composition of cheeses are shown in Table 1.

No significant differences were observed between the cheese trials for the studied proximate composition parameters (*p* > 0.05). Both cheese trials met the requirements for protein source’s products according to Brazilian regulatory standards, which stipulate a minimum content of 10% for food products sources of this nutrient [42]. In terms of fat content, the cheeses in this study were classified as medium-fat according to both Brazilian and Codex Alimentarius standards, as their fat content in dry matter (FDM) fell within the range from 25% to 45% [38,43]. In the present study, the calculated value for total carbohydrates by difference was 9.85 ± 5.34 g/100 g and 10.72 ± 5.28 g/100 g for control and *L. mucosae* cheese, respectively, without significant differences between them (*p* > 0.05). It is worth noting that variable proximate composition values have been reported in the literature for cheeses produced with milk from different species and plant rennet. For instance, Serra et al. [44] conducted a study with cheeses produced from buffalo and sheep milk using kiwi juice as a coagulant and observed ash values ranging from 1.27% to 1.69%, and fat values ranging from 27.40% to 27.55%.

The moisture and acidity results are shown in Table 2.

In terms of moisture content, control and *L. mucosae* cheeses did not differ significantly from each other in the same sampling storage period (*p* > 0.05) and, for both cheeses, their moisture values during storage did not differ significantly (*p* > 0.05). Both control and *L. mucosae* cheeses were classified as high-moisture cheeses in accordance with the Brazilian regulatory standards, with moisture ranging from 46.0% to 54.9% [38]. Moreover, according to the Codex Alimentarius [43], these cheeses were classified as firm/semi-hard, as they exhibited moisture in the free-fat base (MFFB) ranging from 54% to 69%.

Regarding titratable acidity, both cheese trials exhibited a significant increase in acidity during storage at each sampling day (*p* < 0.05). Moreover, the significant difference in acidity between the *L. mucosae* and control cheeses was evident at 30 days and became more pronounced on the final day of storage, with the *L. mucosae* cheese showing twice the acidity of the control product (*p* < 0.05). This increase in acidity can be attributed to the fermentative behavior of *L. mucosae* CNPC007 in goat milk and its acidification capacity in dairy products, as previously demonstrated by this research group [37]. This trend observed especially in *L. mucosae* cheese in the present study is consistent with the findings of Moraes et al. [31], who reported a significant increase in acidity after 14 and 28 days compared to day 1 (*p* < 0.05) for *Coalho* goat cheese manufactured with the commercial Ha-la coagulant (containing protease from *Aspergillus awamori*), *Streptococcus thermophilus* TA40 (a commercial lactic acid culture for dairy applications), and *L. mucosae* CNPC007.

### 3.2. Viability of Lactobacilli during Storage and Its Resistance through the In Vitro Gastrointestinal Conditions

The viability of lactobacilli in *L. mucosae* cheese during storage (mean ± standard deviation) and the survival in this food product when subjected to the in vitro simulated conditions of the gastrointestinal tract (minimum and maximum values) are presented in Table 3.

The highest population of the lactic acid bacteria was observed at 30 days, which differed significantly from the first day (*p* < 0.05). However, no significant difference was observed when comparing the 60th day to the other sampling periods (*p* > 0.05). This indicates that the population of lactobacilli remained relatively stable during the storage period, demonstrating a good viability in the goat cheese with *H. annuus* extract stored at 6 ± 1 °C for 60 days. Similar results were obtained by Moraes et al. [31] in their study with goat Coalho cheese produced using the commercial Ha-la coagulant, *Streptococcus thermophilus* TA40, and *L. mucosae* CNPC007. They reported results ranging from 8.28 to 8.72 log CFU/g for the potentially probiotic autochthonous culture in the product stored at 4 °C for 28 days. According to these authors, the viability of the autochthonous bacteria in the experimental cheeses is aligned with the international recommendations for probiotic foods, which typically require a count of around 10^8^ CFU/g.

Regarding the in vitro assay simulating the gastrointestinal conditions, the results indicate that at the beginning of the assays (0 h), all sampling periods had a nearly similar population of lactobacilli. However, the *L. mucosae* cheeses in all sampling periods exhibited a reduction in the survival of the lactic acid bacteria during the gastric phase (samples collected at 30 min and 2 h). Subsequently, during the enteric phases I and II (samples collected after 4 h and 6 h, respectively), there was an increase in the survival of lactobacilli, indicating partial recovery of viability. Overall, the survival of this bacteria through the in vitro gastrointestinal conditions improved with time. On the first day, only 4.17% of the samples were able to recover lactobacilli above 6 log CFU/g, whereas this percentage increased to 29.17% for samples at 30 and 60 days. For most of the samples analyzed, *L. mucosae* survived at around 5.5 log CFU/g after 4 h of the assay. However, there were exceptions, such as one batch on day 1 and one batch at day 30, where the survival was around 4.01 log CFU/g and 6.56 log CFU/g, respectively. After 6 h of the assay, lactobacilli survived above 6.0 log CFU/g for most samples, except for two batches on day 1 and one batch on day 60. The survival after 6 h of the assay exceeded 7.0 log CFU/g for two batches at 60 days.

The observed behavior, where there was a decrease in the survival of the probiotic during the gastric phase followed by an improvement in the recovered population during the enteric phases, has also been described in other studies [37,45]. According to these studies, the decrease in the population of lactobacilli in the gastric phase is attributed to the stress caused by the decrease in pH, which leads to injury to the microorganisms. However, this decrease is typically reversed in the enteric phase due to the increase in pH, which allows for the repair and recovery of the microorganisms.

Based on the results, it is evident that lactobacilli maintained good viability throughout the entire storage period in the goat cheeses with *H. annuus* extract. Additionally, this microorganism exhibited satisfactory survival in the cheese when subjected to the in vitro simulated gastrointestinal conditions, as it was recoverable at the end of the assays.

### 3.3. Concentration of Microbial Contamination Indicators in Cheeses during Storage

The results of the analysis of the microbial contamination indicators in the cheeses are shown in Table 4. The results of total coliforms (at 35 °C), thermotolerant coliforms (at 45 °C), and *E. coli* are presented as the most probable number (MPN)/g sample, while the results of *Staphylococcus* spp. are presented as CFU/g of sample.

All cheeses were negative for the presence of *Salmonella* spp. in 25 g of product and exhibited results of *E. coli* and thermotolerant coliforms below the method threshold (<3 MPN/g). The total coliform counts decreased in all batches, except for one batch of the control cheese. All cheeses met the acceptable quality criteria for *E. coli* and *Salmonella* spp. according to the Brazilian regulatory standards in effect at the time of the study [39], which specified a maximum population of 10^2^ MPN/g for *E. coli* in cheeses with moisture of 46% or above and the absence of *Salmonella* spp./25 g of product. *Staphylococcus* spp. was detected in one batch of control cheese on the 1st and 30th days of storage (9.5 × 10³ CFU/g and 4.0 × 10^2^ CFU/g, respectively), leading to an unsatisfactory result in terms of this parameter’s quality, as it exceeded the maximum limit established for this microorganism in cheeses by the same Brazilian regulatory standards, which was 10^2^ CFU/g [39]. However, by the 60th day, *Staphylococcus* spp. count had decreased below the detectable threshold the methodology (<10^2^ CFU/g).

Based on these results, the control trial cheeses, without *L. mucosae* CNPC007, were more susceptible to contamination with total coliforms and *Staphylococcus* spp., while these contaminants were reduced or prevented in the batches from the probiotic trial.

The results of *Staphylococcus* spp. falling below the detectable threshold of the methodology and the higher reduction in the number of total coliforms in the cheeses containing *L. mucosae* at 60 days of storage may indicate a potential inhibitory effect of this microorganism on these microbial indicators, contributing to the biopreservation of the product during storage. This effect is most probably due to the higher values of titratable acidity over the storage. The biopreservative effect of potentially probiotic lactic acid bacteria has been reported in other studies. Buriti, Cardarelli, and Saad [46] conducted a study involving the addition of a coculture of *Lacticaseibacillus paracasei* LBC82 (formerly *Lactobacillus paracasei* LBC82) and *Streptococcus thermophilus* TA40 to fresh cream cheeses. In this study, total coliforms were initially observed at levels between 1.0 and 1.4 log CFU/g of sample (equivalent to 10 and 25 CFU/g) on the day of production (day 0). However, as the study progressed, the number of coliforms in these cheeses decreased to levels below the method’s threshold starting from day 14. Additionally, the authors confirmed that *L. paracasei* exhibited inhibitory effects against *Latilactobacillus sakei* (formerly *Lactobacillus sakei*), *Listeria monocytogenes*, and *Staphylococcus aureus* when tested in MRS agar, where inhibition zones measuring between 2 and 3 cm were observed. Recently, Galdino et al. [33] verified that *L. mucosae* CNPC007, when tested in vitro in Mueller-Hinton agar, was able to inhibit *E. coli* ATCC 25922, *Salmonella* Typhimurium ATCC 14028, and *S. aureus* ATCC 25923 by, respectively, 30.00%, 20.87%, and 23.21% of the inhibition capacity of the positive control Ciprofloxacin 2 mg/mL.

### 3.4. Proteolytic Analysis by Sodium Dodecyl Sulphate Polyacrylamide Gel Electrophoresis (SDS-PAGE)

**Figure 2:**
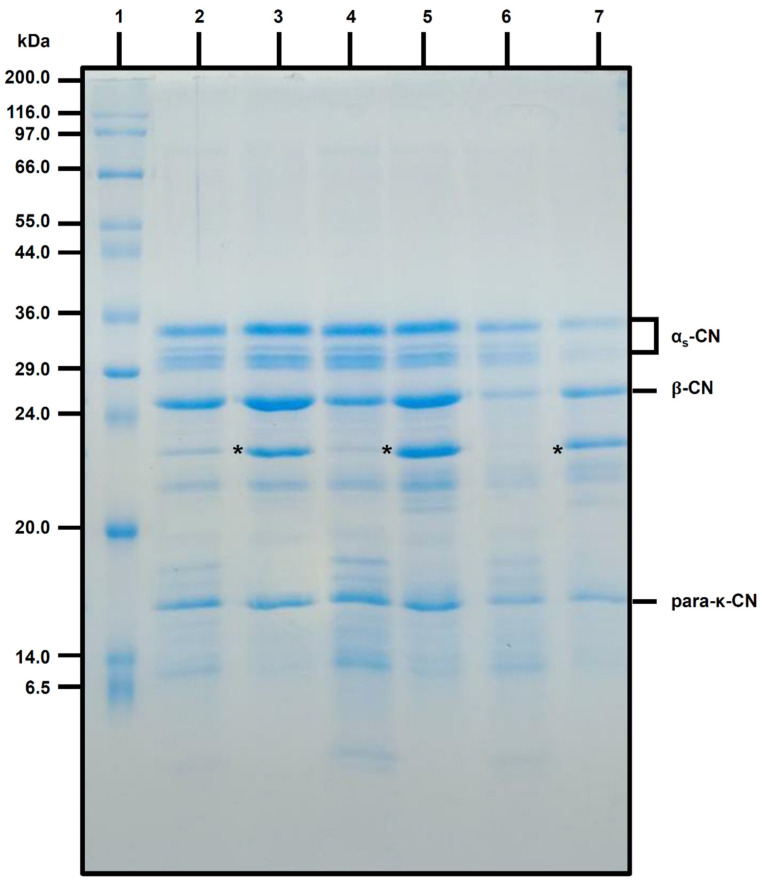
Sodium dodecyl sulfate polyacrylamide gel electrophoresis of control and *L. mucosae* cheeses in the day of packing (day 1) and after 30 and 60 days of storage at 6 ± 1 °C. The SDS-PAGE pattern of standard mixture Sigma Marker (Wide Range 6500–200,000 Da, Sigma-Aldrich) is shown in lane 1. The pattern of control cheeses on days 1, 30, and 60 is shown in lanes 2, 4 and 6, respectively, while the pattern of *L. mucosae* cheeses is shown in lanes 3, 5, and 7 for the same sampling periods, respectively. αs-CN = αs-casein. β-CN = β-casein. para-κ-CN = para-κ-casein, * = highlight for the intense bands verified in *L. mucosae* cheeses.

The SDS-PAGE electrograms of one batch of control and *L. mucosae* cheeses after 1, 30, and 60 days of storage are shown in Figure 2.

In all treatments, characteristic cheese protein and peptide bands were observed, with a notable presence of caseins (CN), particularly α_s_-CN and β-CN, as well as the para-κ-CN peptide. On days 1 and 30, the revealed protein and peptide bands in *L. mucosae* cheese appeared more intense than those in the control cheeses.

**Figure 3:**
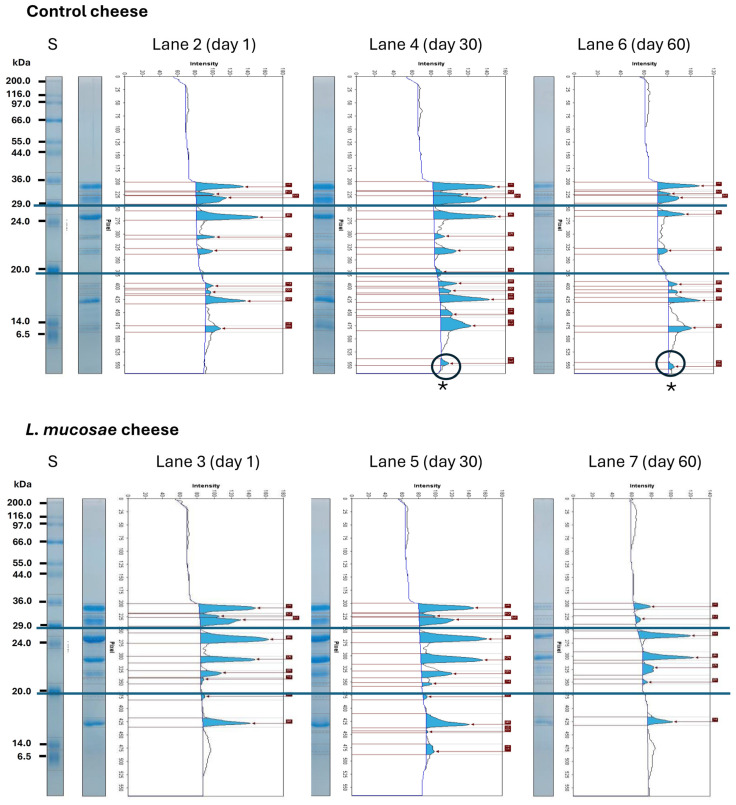
Densitometric analysis of bands obtained by SDS-PAGE for cheeses at 1, 30, and 60 days of storage of control (lanes 2, 4, and 6, respectively) and *L. mucosae* (lanes 3, 5, and 7, respectively) trials. The SDS-PAGE pattern of standard mixture Sigma Marker (Wide Range 6500–200,000 Da, Sigma-Aldrich) is labelled as “S”. * = highlight for the prominent peaks marked with a circle in the region below 6.5 kDa in control cheeses at 30 and 60 days.

**Figure 4:**
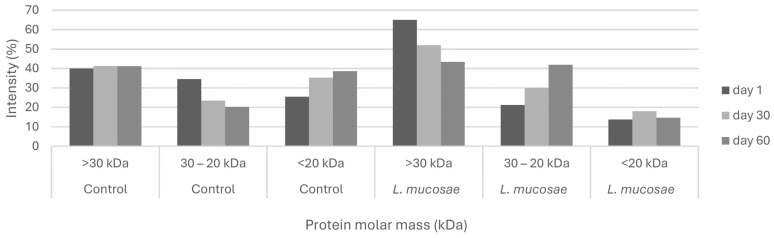
Relative intensity (%) of proteins obtained by SDS-PAGE and quantified by densitometric analysis using GelAnalyzer 23.1.1 in control and *L. mucosae* cheeses after 1, 30, and 60 days of storage.

In order to evaluate the protein distributions between cheese trials and their mobility during the storage, the densitometric analysis of the SDS-PAGE gel was performed (Figure 3) and, for a better discussion of these densitometric results, protein bands were divided into three groups, according to their molecular weights: >30 kDa, 30–20 kDa, and <20 kDa. Areas of density peaks provided the relative proportion (%) of proteins, shown in Figure 4, which were used to evaluate their changes and proteolysis during storage in control and *L. mucosae* cheeses.

At 1 and 30 days of storage, *L. mucosae* cheeses showed higher relative proportions of >30 kDa proteins compared to control cheeses (Figure 4). However, by day 60, there was a tendency for the intensity of these bands to decrease in *L. mucosae* cheeses. In control cheeses, on the other hand, the relative proportion of >30 kDa proteins remained stable during all the storage. For the relative proportion of 30–20 kDa proteins, an opposite behavior was verified, with a decrease of this group of proteins during storage in control cheeses, and with an increase in *L. mucosae* cheeses. Considering the proteins of this range in the Figure 2, for all storage periods, there were prominent bands, marked with “*” in the region below 24 kDa in *L. mucosae* cheeses, which were either poorly visible (days 1 and 30) or not visible (60 days) in the control cheeses. Conversely, in the control cheese at all sampling periods, the bands in the 6.5 to 14 kDa range were more intense (Figure 2), parallel to the higher relative proportions of <20 kDa proteins (Figure 4), compared to their respective *L. mucosae* cheeses, with the control cheese also exhibiting increase in the relative proportion of these proteins during storage. Additionally, prominent peaks marked with a circle and “*” in the region below 6.5 kDa in the densitograms were observed only in control cheeses at 30 and 60 days (Figure 3).

Therefore, in the present study, a distinct pattern of proteolysis was observed in control and *L. mucosae* cheeses, with the relative proportions of 30–20 kDa proteins decreasing at the end of storage, with an increase of the <20 kDa proteins and <6.5 kDa peptides, while for *L. mucosae* cheeses, the proteolysis during storage was verified for >30 kDa proteins, with an increase in 30–20 kDa proteins at 60 days. These changes were more evident at the end of storage for both cheeses. According to Barać et al. [47] several factors, including the residual rennet, the indigenous milk proteases, and also the starter and non-starter microbiological cultures, are responsible for proteolysis obtained in cheese. In this study, at 60 days, the tendency for the mobility of 30–20 kDa proteins to <20 kDa bands was probably due to the residual plant rennet from sunflower seed extract, while in *L. mucosae* cheeses, the proteolysis of >30 kDa proteins was probably due to the lactic acid bacteria metabolism. Even with this proteolytic pattern in *L. mucosae* cheeses, the presumable amino acid degradation was insufficient to interfere in the titratable acidity of the product due also to the high lactic acid production verified in cheeses containing this bacterium.

Regarding the proteolytic potential of plant extracts, a study conducted by Egito et al. [17] using extracts of sunflower and Albizia (*Albizia lebbeck*) seeds, as analyzed through electrophoresis and high-performance liquid chromatography, revealed that α_s_-CN and β-CN were more susceptible to hydrolysis by Albizzia extract compared to sunflower extract. In the presence of the Albizia extract, most of the α_s_-CN disappeared after 40 min, while the band of β-CN was still visible after 6 h. In the presence of sunflower extract, both α_s_-CN and β-CN remained visible after 6 h, with traces of these caseins still detected after 24 h.

Moreover, the regions where α_s_-CN, β-CN and para-κ-CN bands were revealed in the gels for both control and *L. mucosae* cheeses in the present study (Figure 2) closely resembled the regions where these same bands appeared in the study conducted by Egito et al. [17]. However, as previously mentioned, the protein profile of *L. mucosae* cheese differed from that of the control cheese also due to the presence of a band with strong intensity between 20 and 24 kDa.

As observed in the present study for the proteolysis of cheeses with *L. mucosae* strain CNPC007, the proteolytic potential of this microorganism has already been described in some studies, such as Morais et al. [48], where a greater proteolytic extension was reported in yogurt containing *L. mucosae* compared to a control yogurt without the culture, and Moraes et al. [30], who described the possible proteolysis of *L. mucosae* in goat cheeses as a potential source of aroma alteration throughout the cheese maturation period.

## 4. Conclusions

Goat cheese processed with *H. annuus* seed extract as plant rennet is a promising option as *L. mucosae* CNPC007 carrier, since this microorganism showed either high viability and stability in the product during storage or high survival under in vitro gastrointestinal conditions. The biochemical properties of the cheese were significantly influenced either by the *H. annuus* seed extract, an alternative source of proteases to animal and microbial rennet that allowed protein breakdown and release of peptides in control cheese, or by *L. mucosae* CNPC007 that resulted in a product with distinct proteolytic profile, allied to its potential bioprotective effect, probably as a result of the acidification capacity of this lactic acid bacteria.

## Figures and Tables

**Table 1:** Proximate composition of cheeses (mean ± standard deviation).

Item	Cheese Trial	
	Control	*L. mucosae*
Total solids (g/100 g) *	52.65 ± 2.80 ^A^	52.86 ± 1.39 ^A^
Ash (g/100 g)	3.74 ± 0.23 ^A^	3.65 ± 0.38 ^A^
Fat (g/100 g)	21.79 ± 1.96 ^A^	22.72 ± 1.62 ^A^
FDM (g/100 g)	41.61 ± 5.60 ^A^	42.95 ± 2.38 ^A^
MFFB (g/100 g)	60.63 ± 4.77 ^A^	60.99 ± 1.25 ^A^
Protein (g/100 g)	19.09 ± 2.69 ^A^	17.49 ± 1.50 ^A^
Total carbohydrates (g/100 g)	9.85 ± 5.34 ^A^	10.72 ± 5.28 ^A^

* Average of sampling days 1, 30, and 60. FDM, fat in dry matter. MFFB, moisture in the free-fat base. ^A^, superscript capital letter in the same row denotes that the trials did not differ significantly from each other (*p* < 0.05).

**Table 2:** Moisture and acidity values during the cheese storage time (mean ± standard deviation).

Parameter			Time (Days)	
	Trial	1	30	60
Moisture (g/100 g)	Control	46.78 ± 4.16 ^Aa^	47.68 ± 2.14 ^Aa^	47.59 ± 1.72 ^Aa^
	*L. mucosae*	46.73 ± 2.66 ^Aa^	46.98 ± 2.62 ^Aa^	47.70 ± 0.62 ^Aa^
Acidity	Control	0.39 ± 0.097 ^Aa^	0.60 ± 0.040 ^Ab^	0.97 ± 0.23 ^Ac^
(g lactic acid/100 g)	*L. mucosae*	0.50 ± 0.059 ^Aa^	1.04 ± 0.46 ^Bb^	1.80 ± 0.08 ^Bc^

^A, B^ different superscript capital letters in a column denote that the trials differed significantly from each other (*p* < 0.05). ^a, b, c^ different superscript lowercase letters in a row denote that the storage times differed significantly from each other for a same trial (*p* < 0.05).

**Table 3:** Viability of lactobacilli in cheeses during the storage period at 6 ± 1 °C (mean ± standard deviation, log CFU/g) and its survival in the product when submitted through the in vitro the gastrointestinal resistance assay (minimum–maximum values, log CFU/g).

			**Time (Days)**			
	1		30		60	
	**Mean ± Standard Deviation (log CFU/g)**					
Viability	8.43 ± 0.29 ^a^		8.76 ± 0.12 ^b^		8.75 ± 0.24 ^a,b^	
	**Range**					
	Min.–Max. (log CFU/g)	% above 6 log CFU/g	Min.–Max. (log CFU/g)	% above 6 log CFU/g	Min.–Max. (log CFU/g)	% above 6 log CFU/g
Before the assay—0 h	8.66–9.05	100 (6/6)	8.49–8.85	100 (6/6)	8.75–9.05	100 (6/6)
During the assay						
30 min	3.64–5.95	0 (0/6)	2.90–6.30	16.66 (1/6)	5.77–6.47	33.33 (2/6)
2 h	<LT–5.00	0 (0/6)	4.94–5.60	0 (0/6)	5.03–5.63	0 (0/6)
4 h	<LT–5.83	0 (0/6)	5.36–6.58	33.33 (2/6)	5.00–5.90	0 (0/0)
6 h	<LT–6.06	16.66(1/6)	<LT–7.05	66.66 (4/6)	5.22–7.54	83.33 (5/6)
Assay (overall range)	<LT–6.06	4.17 (1/24) ^a^	<LT–7.05	29.17 (7/24) ^b^	5.00–7.54	29.17 (7/24) ^b^

^a, b^_,_ different superscript lowercase letters in a row denote that the storage times differed significantly from each other (*p* < 0.05) for the viability of lactobacilli in cheese during storage or that the storage times for the overall in vitro assay consolidated results differed significantly from each other in the exact binomial test (*p* < 0.05) for the viability rate above 6.00 log cfu/g, the probiotic amount considered relevant to result in a beneficial effect to the consumer, considering the null hypothesis (h_0_) that this proportion during the passage through the in vitro simulation of the gastrointestinal tract was at least 25%. LT = lower than method threshold.

**Table 4:** Populations of microbial contaminants detected in the three batches of control and *L. mucosae* cheeses during the storage at 6 ± 1 °C.

		Bacterial Count Range			% of Samples above the Threshold (No. of Above the Threshold/Total No. Analysed)		
Microbial contaminant	Trial		Time (days)			Time (days)	
		1	30	60	1	30	60
Total coliforms (at 35 °C, MPN/g)	Control	3.6–>1100	<3–>1100	<3–>1100	100 (3/3) ^A^	66.7 (2/3) ^A^	66.7 (2/3) ^B^
	*L. mucosae*	>1100	93–>1100	<3	100 (3/3) ^A^	100 (3/3) ^A^	0 (0/3) ^A^
Thermotolerant coliforms (at 45 °C, MPN/g)	Control	<3	<3	<3	0 (3/3) ^A^	0 (3/3) ^A^	0 (3/3) ^A^
	*L. mucosae*	<3	<3	<3	0 (3/3) ^A^	0 (3/3) ^A^	0 (3/3) ^A^
*E. coli* (MPN/g)	Control	<3	<3	<3	0 (3/3) ^A^	0 (3/3) ^A^	0 (3/3) ^A^
	*L. mucosae*	<3	<3	<3	0 (3/3) ^A^	0 (3/3) ^A^	0 (3/3) ^A^
*Salmonella* spp. (in 25 g)	Control	Absent	Absent	Absent	0 (3/3) ^A^	0 (3/3) ^A^	0 (3/3) ^A^
	*L. mucosae*	Absent	Absent	Absent	0 (3/3) ^A^	0 (3/3) ^A^	0 (3/3) ^A^
*Staphylococcus* spp. (CFU/g)	Control	<10^2^–9.5 × 10^3^	<10^2^–4.0 × 10^3^	<10^2^	33.3 (1/3) ^B^	33.33 (1/3) ^B^	0 (0/3) ^A^
	*L. mucosae*	<10^2^	<10^2^	<10^2^	0 (3/3) ^A^	0 (3/3) ^A^	0 (0/3) ^A^

^A^, ^B^, different superscript capital letters in a column denote that cheese trials differed significantly from each other (*p* < 0.05) for the same microbial indicator in the same sampling period.

## Data Availability

The original contributions presented in the study are included in the article/Supplementary Materials, further inquiries can be directed to the corresponding author.

## Supplementary Materials

The following supporting information can be downloaded at: https://www.mdpi.com/article/10.3390/foods13182905/s1, Figure S1. Appearance of *Helianthus annuus* (sunflower) seed extract used in the present study; Figure S2. Coagulant activity in three test tubes containing 40 μL *Helianthus annuus (sunflower)* seed extract and 1 mL of reconstituted milk powder after 1 h.
