# Supplementary material for: Goat Cheese Produced with Sunflower (Helianthus annuus L.) Seed Extract and a Native Culture of Limosilactobacillus mucosae: Characterization and Probiotic Survival
Source: Foods. 2024 Sep 13;13(18):2905. doi: 10.3390/foods13182905 (PMC11431575; doi:10.3390/foods13182905)
Supplement: Supplementary file 1 [file foods-13-02905-s001.zip › foods-3060538-supplementary.pdf]

Supplementary Material

Goat Cheese Produced with Sunflower (*Helianthus annuus* L.) Seed Extract and a Native Culture of *Limosilactobacillus mucosae*: Characterization and Probiotic Survival

Dôrian Cordeiro Lima Júnior [1,2], Viviane Maria da Silva Quirino [2], Alícia Santos de Moura [2], Joyceana Oliveira Correia [1,2], João Ricardo Furtado [3], Isanna Menezes Florêncio [2], Márcia Maria Cândido da Silva [4], Hévila Oliveira Salles [3], Karina Maria Olbrich dos Santos [5], Antonio Silvio do Egito [4,*] and Flávia Carolina Alonso Buriti [1,2]

[1] Programa de Pós-Graduação em Ciências Farmacêuticas, Universidade Estadual da Paraíba, R. Juvêncio Arruda, s/n, Campina Grande 58429-600, PB, Brazil; mrjuniorc7@gmail.com (D.C.L.J.); joyceanaoliveira@gmail.com (J.O.C.); flavia@servidor.uepb.edu.br (F.C.A.B.)

[2] Núcleo de Pesquisa e Extensão em Alimentos, Universidade Estadual da Paraíba, R. Juvêncio Arruda, s/n, Campina Grande 58429-600, PB, Brazil; qviviane9@gmail.com (V.M.d.S.Q.); aliciamoura1998@gmail.com (A.S.d.M.); isannamenezes@hotmail.com (I.M.F.)

[3] Embrapa Caprinos e Ovinos, Estrada Sobral/Groaíras, km 4, Sobral 62010-970, CE, Brazil; joao.furtado@embrapa.br (J.R.F.); hevila.salles@embrapa.br (H.O.S.)

[4] Embrapa Caprinos e Ovinos, Núcleo Regional Nordeste, R. Osvaldo Cruz, 1143, Campina Grande 58428-095, PB, Brazil; marciamcandido@gmail.com

[5] Embrapa Agroindústria de Alimentos, Av. das Américas, 29501, Rio de Janeiro 23020-470, RJ, Brazil; karinal.dos-santos@embrapa.br

* Correspondence: antoniosilvio.egito@embrapa.br

Supplementary

Helianthus annuus *(sunflower) seed extract*

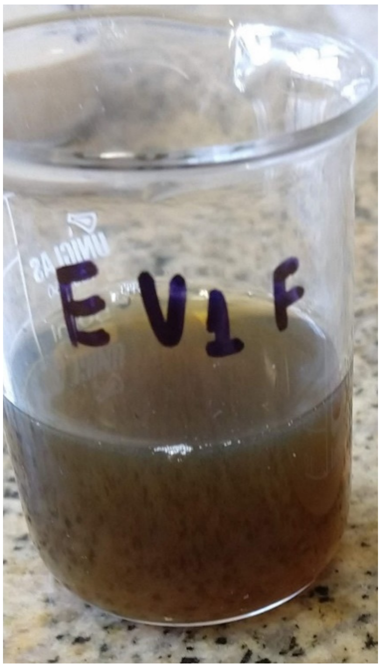

Figure S1. Appearance of *Helianthus annuus* (sunflower) seed extract used in the present study.

Coagulant activity of the Helianthus annuus *(sunflower) seed extract*

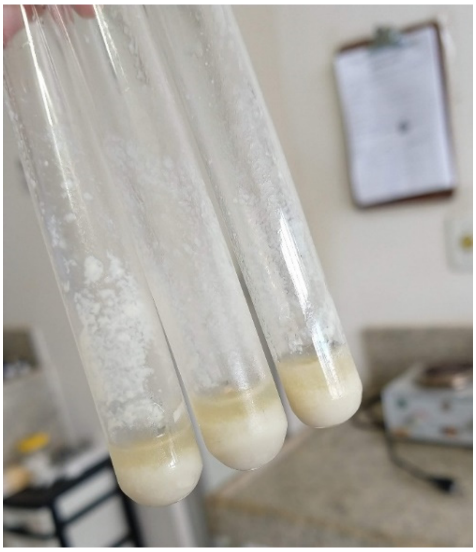

Figure S2. Coagulant activity in three test tubes containing 40 µL *Helianthus annuus (sunflower)* seed extract and 1 mL of reconstituted milk powder after 1 h.
